# Dengue virus-free defective interfering particles have potent and broad anti-dengue virus activity

**DOI:** 10.1038/s42003-021-02064-7

**Published:** 2021-05-11

**Authors:** Dongsheng Li, Min-Hsuan Lin, Daniel J. Rawle, Hongping Jin, Zhonglan Wu, Lu Wang, Mary Lor, Mazhar Hussain, John Aaskov, David Harrich

**Affiliations:** 1 Department of Cell and Molecular Biology, QIMR Berghofer Medical Research Institute, Herston, QLD Australia; 2 Ningxia Center for Disease Control and Prevention, Ningxia, China; 3 Institute of Health and Biomedical Innovation, Queensland University of Technology, Brisbane, QLD Australia

**Keywords:** Microbiology, Molecular biology

## Abstract

Dengue virus (DENV) is spread from human to human through the bite of the female *Aedes aegypti* mosquito and leads to about 100 million clinical infections yearly. Treatment options and vaccine availability for DENV are limited. Defective interfering particles (DIPs) are considered a promising antiviral approach but infectious virus contamination has limited their development. Here, a DENV-derived DIP production cell line was developed that continuously produced DENV-free DIPs. The DIPs contained and could deliver to cells a DENV serotype 2 subgenomic defective-interfering RNA, which was originally discovered in DENV infected patients. The DIPs released into cell culture supernatant were purified and could potently inhibit replication of all DENV serotypes in cells. Antiviral therapeutics are limited for many viral infection. The DIP system described could be re-purposed to make antiviral DIPs for many other RNA viruses such as SARS-CoV-2, yellow fever, West Nile and Zika viruses.

## Abstract (web summary)

Li et al. produce a dengue virus (DENV)-derived defective interfering particle (DIP) production cell line that can stably produce infectious virusfree DIPs. The DENV DIPs produced from this system can potently inhibit replication of all DENV serotypes in cells and have the potential to be repurposed to make antiviral DIPs for other RNA viruses.

## Introduction

Dengue virus (DENV) is a member of the genus Flavivirus, as is West Nile virus, yellow fever virus, and Zika virus, with a small positive strand RNA genome. There are four serotypes, DENV-1 to -4, that are transmitted by mosquitoes and each year cause ~100 million clinical infections, about 0.5 million hospitalizations and 25,000 deaths. Currently, antiviral drugs are not available for clinical use. The first licensed dengue vaccine is available but its use and effectiveness are limited^1^.

DIPs are defective virions discovered more than 70 years ago in populations of influenza A (IAV) viruses^2^ and have been reported for almost every class of RNA virus including alphaviruses^3,4^, coronaviruses^5–7^, and flaviviruses^8–10^. DIPs have been found in laboratory cultures, in virus infected animals and patients^8,11–15^. DIPs are defined as virus-like particles that: (i) contain a normal set of viral proteins; (ii) contain a partial parental viral genome, which is referred to as a DI genome or DI RNA; (iii) require a parental wild type (WT) helper virus to reproduce; and (iv) interfere specifically with the intracellular replication of the parental virus^16^. DIPs inhibit virus replication in a dose-dependent manner in cell culture^17^, and interference with viral replication in vivo by natural DIPs has been inferred in animal infections by a reduction in disease severity and reduced viral titers^18,19^. DIPs are transmitted with and are believed to co-evolve with WT virus so as to continue to inhibit virus replication^20^.

A hallmark of DIPs is that their production requires a WT helper virus that provides all of the necessary viral enzymes, co-factors and RNA packaging mechanisms so that DI RNA can replicate, be assembled into DIPs that will be secreted by infected cells^16^. Recently a system to produce IAV-derived DIPs that are free of infectious virus was described^21^. The system used a combination of cell lines stably expressing IAV PB2, a subunit of the viral RNA polymerase, that were transiently transfected with a plasmid that expressed the IAV DI RNA 244-mScarlet-I, which could inhibit replication of different IAV strains^16^.

How DIPs reduce WT virus replication from which they are derived has not been fully elucidated. DIPs parasitize cellular and viral resources required by WT viruses replication^13^, and may stimulate cellular innate antiviral responses^22^. For these reasons, DIPs have potential to be employed as antiviral agents^22^. However, the use of DIPs as therapy or prophylaxis has been hampered by DIP preparations that are contaminated by infectious helper virus that is impractical to remove.

This study reports a novel DENV DIP production cell line that can stably produce infectious virus-free DIPs using a combination of lentiviral and retroviral vectors. The DENV DIPs produced from this system can potently inhibit replication of all four DENV serotypes in cells.

## Results

### DENV-free DIPs produced by a cell line

**Fig. 1:**
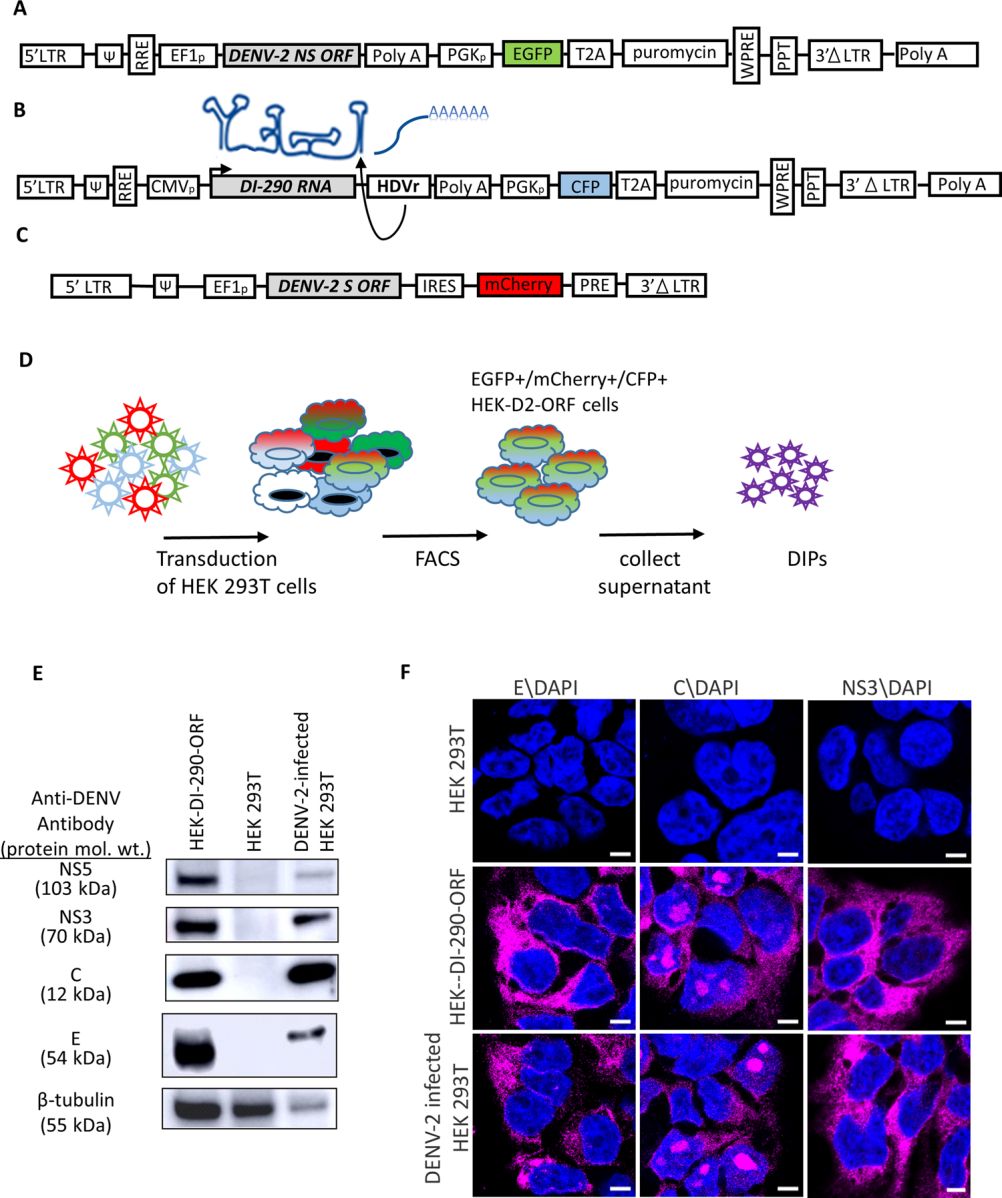
Establishment of a DENV DIP production cell line using HEK 293T cells. **A** A lentiviral vector expressing the DENV-2 NS proteins and EGFP. **B** A lentiviral vectors that expresses the DI-290 RNA as an mRNA that is processed by the HDVr into authentic DI-290 RNA and expresses CFP. **C** A retroviral vector that expresses DENV-2 S proteins and mCherry. **D** The transduction of HEK 293T cells can be selected by FACS for triple-positive cells expressing EGFP, mCherry and CFP. **E** Western blot of HEK-DI-290-ORF cells using antibodies to DENV NS5, NS3, C and E proteins. The relative protein loading in each lane is indicated by a western blot for β-tubulin. **F** Confocal fluorescence microscopy of HEK 293T, DENV-2 infected HEK 293T, and HEK-DI-290-ORF cells stained with antibodies to DENV E, C, and NS3 (magenta). Cells were counter stained with DAPI (blue). The white bar in the image represents 10 µm. mol. wt. is molecular weight.

A tri-vector approach was used to develop a cell line that continuously produces infectious virus-free DENV DIPs (Fig. 1). Two SIN-type lentiviral vectors were used to express DENV-2 non-structural (NS) proteins NS1 to NS5 (Fig. 1a) and a 290 nucleotide (nt) DI RNA originally identified in people infected by DENV-2 that will be hereafter referred to as DI-290 RNA (Fig. 1b)^9^. A SIN-type retroviral vector was used to express the DENV-2 structural (S) proteins capsid (C), membrane (prM), and envelope (E) (Fig. 1c). The S and NS sequences were codon optimized in order to improve protein expression. A hepatitis D virus ribozyme was positioned downstream of DI-290 RNA in order to cleave the correct 3′ end^23^, which has been used previously to make infectious DENV RNA from a bacterial artificial chromosome in cells^24^. The HEK 293T cells were transduced with all three vectors and cells that expressed the fluorescent reporter proteins enhanced green fluorescent protein (EGFP), mCherry and cyan fluorescent protein (CFP) were selected by fluorescence-activated cell sorting (FACS) (Fig. 1d). This cell line was called HEK-DI-290-ORF. A Vero E6 (Vero) cell line also was prepared that expressed only DENV-2 S and NS protein (Vero-ORF, Supplementary Fig. S1) and an HEK 293T cell line that expressed only the DI-290 RNA was designated as HEK-DI-290.

The expression of DENV proteins E, C, NS3, and NS5 was detected in lysates made from HEK-DI-290-ORF cells by western blot (Fig. 1e), and was consistent with high levels of S and NS mRNA detected by RT-qPCR to detect E, NS1 and NS5 (Supplementary Fig. S2). The subcellular distribution of C, E and NS3 proteins in HEK-DI-290-ORF cells, detected by confocal fluorescence microscopy, was similar to that in cells infected with DENV 2 (Fig. 1f). DENV C also was also observed in nucleolar regions of HEK-DI-290-ORF and DENV-2 infected HEK 293T cells^25,26^.

### DI RNA replication in HEK-DI-290-ORF cells

**Fig. 2:**
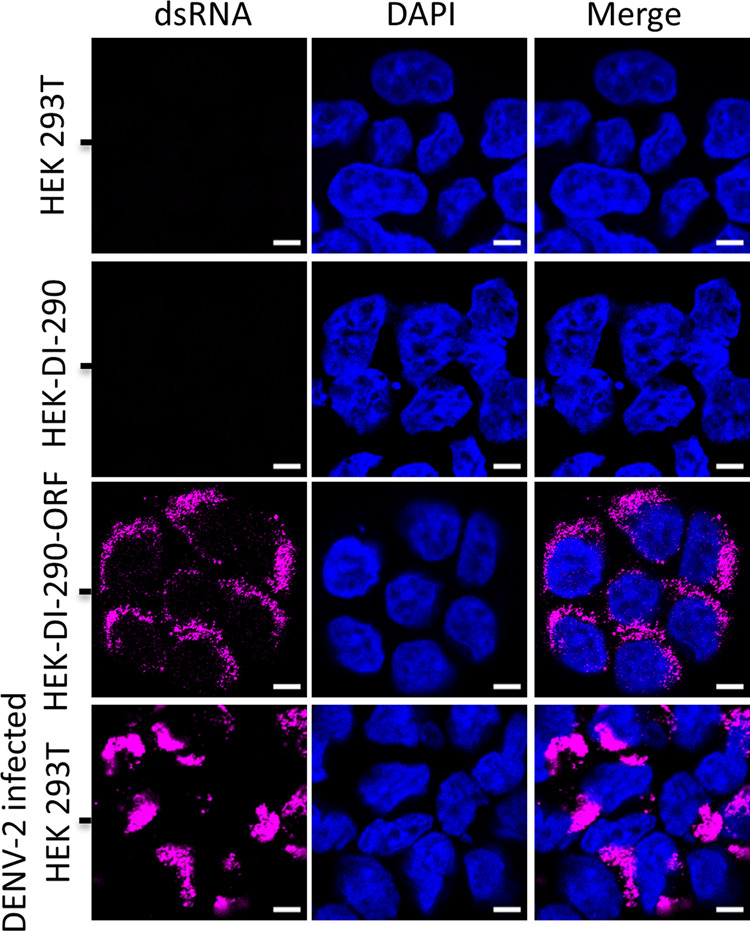
DI-290 RNA replication in HEK-DI-290-ORF cells. Confocal fluorescence microscopy of HEK 293T, HEK-DI-290, HEK-DI-290-ORF, and DENV-2 infected HEK 293T cells that were stained with an anti-double strand RNA antibody (magenta). The cells were counterstained with DAPI to stain nuclei (blue). The white bar in the image represents 10 µm.

DENV is a positive strand RNA virus that must synthesize negative strand viral RNA and form double strand viral RNA intermediates in order to replicate the viral RNA genome^27,28^. Confocal fluorescence microscopy of HEK-DI-290-ORF cells detected double strand RNA (Fig. 2, third row) and in DENV-2 infected HEK 293T cells (Fig. 2, fourth row), indicating that RNA replication was occurring. As expected, double strand RNA was not observed in HEK 293T cells and HEK-DI-290 cells (Fig. 2, first and second rows). The results indicates that DI-290 RNA replicated in HEK-DI-290-ORF cells.

### HEK-DI-290-ORF cells produce a virus-like particles that associate with DI-290 RNA

**Fig. 3:**
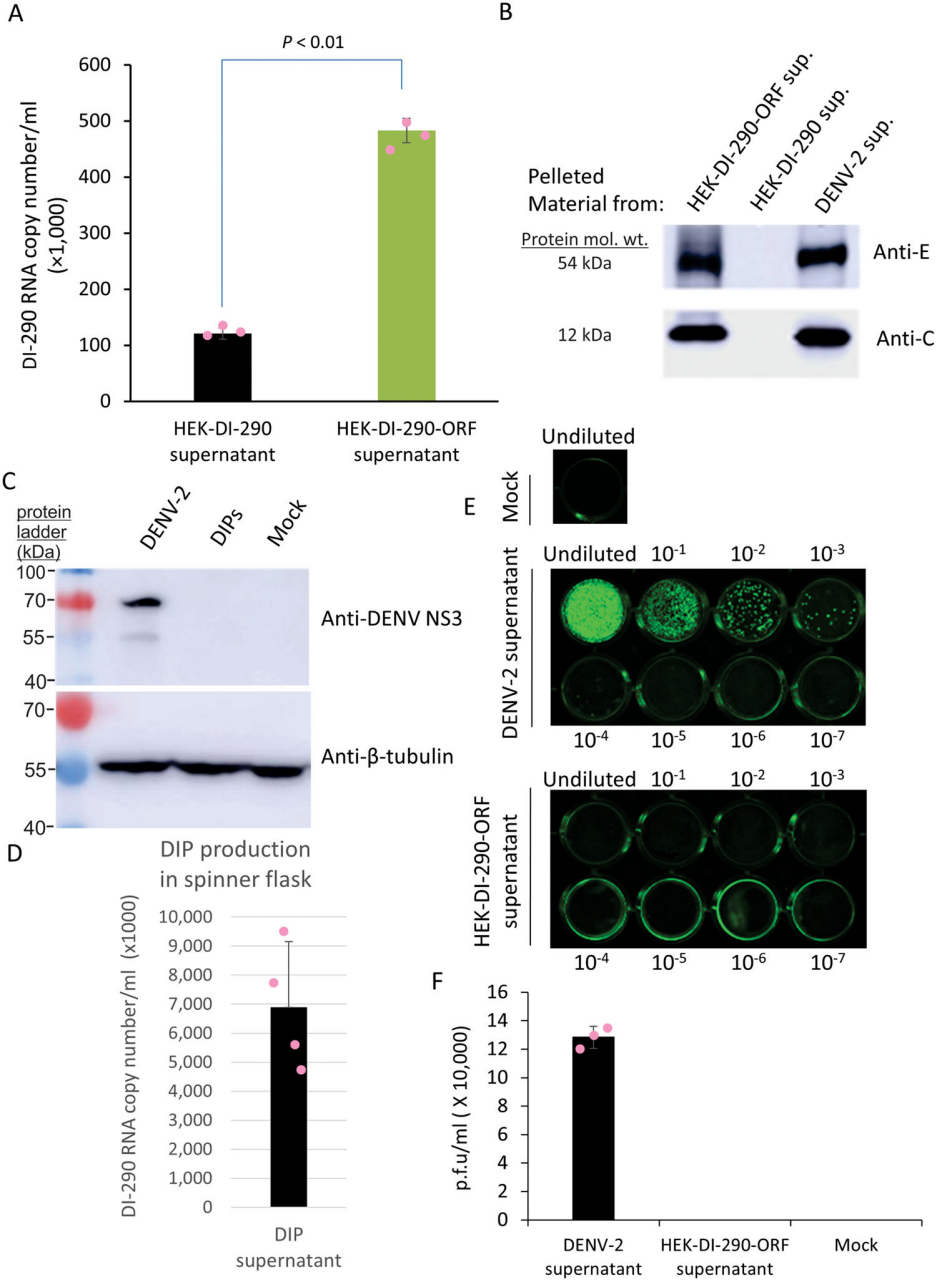
Evidence that HEK-DI-290-ORF cells make infectious virus-free DIPs. **A** Triplicate samples of cell-free culture supernatant of HEK-DI-290 or HEK-DI-290-ORF cells underwent ultracentrifugation. The pelleted material was assayed by RT-qPCR to measure the level of DI-290 RNA. The mean value, SD and calculated a *P* value is shown. (**B**) Duplicates of the pelleted samples from **A** were assayed by western blot using and anti- DENV-2 E or anti-DENV-2 C antibodies. The pelleted material of cell-free culture supernatant of DENV-2 infected cells were used as a positive control. **C** Vero cells were incubated with DENV-2 using an MOI of 0.01 or with culture supernatant from HEK-DI-290-ORF cells. Lysates were made after 7 days and assayed by western blot using an anti-DENV NS3 antibody, or with anti-B-tubulin to monitor the amount of protein loaded in each lane. **D** HEK-DI-290-ORF cells were grown in a glass spinner flask using serum-free medium. The supernatant was filtered (0.45 µM), nuclease treated and DIPs were pelleted at 100,000 × *g*. RNA was extracted from the pelleted material and assayed by RT-qPCR to measure the level of DI-290 RNA in the sample. The mean value of DI-290 RNA measured in copies/ mL and SD from four independent cultures is shown. **E** DENV-2 supernatant (equivalent to 3 × 10^7^ NS5 RNA copies) and culture supernatant from HEK-DI-290-ORF cells (equivalent to 3 × 10^7^ DI-290 RNA copies) were tenfold serially diluted and incubated with Vero cells. The plates were fixed with acetone/methanol 5 days later and plaques were visualized by staining with an anti-Flavivirus E antibody. **F** Plaques were counted to determine virus titer 5 days post-infection. Data shown are mean values ± SD from three replicate experiments. mol. wt. is molecular weight.

Culture supernatant from HEK-DI-290-ORF cells and from HEK-DI-290 cells was clarified and filtered to remove cells, treated with nuclease and then centrifuged at 100,000 × *g* at 4 °C for 60 min. RNA was extracted from the pelleted material and DI-290 RNA was quantified by RT-qPCR. The amount of DI-290 RNA from pelleted material of HEK-DI-290-ORF cell supernatant was 4-fold higher than a supernatant from HEK-DI-290 cells (Fig. 3a) (Supplementary Data 1), indicating the presence of high density complexes and/or virus-like particles (VLP) containing DI-290 RNA in the culture supernatant of both cell lines.

Western blot analysis of material pelleted from the filtered supernatant of cultures of HEK-DI-290-ORF, HEK-DI-290, and DENV-2 infected HEK 293T cells identified E and C proteins in material from HEK-DI-290-ORF and DENV-2 infected cell supernatants, but not from HEK-DI-290 supernatant (Fig. 3b). The data indicates that HEK-DI-290-ORF cells produce DENV-2-derived VLPs that contain viral structural proteins and DI RNAs, which are characteristics of DIPs.

As DI-290 RNA also was detected in pelleted material from culture supernatant of HEK-DI-290 cells, an immune-depletion experiment was performed to determine if DI-290 RNA might be associated with exosomes. Exosomes are extracellular vesicles that can contain cell-derived small RNAs, proteins, lipids and CD63 as a marker^29^. HEK-DI-290-ORF and HEK-DI2-90 cell culture supernatants were incubated with an anti-CD63 antibody coupled to Dynabeads to bind exosomes and RNA was extracted from depleted supernatants. Levels of DI-290 RNA detected by RT-qPCR were reduced in the HEK-DI-290 sample when exosomes were depleted, while this treatment had no effect on DI-290 RNA levels in the DIP sample (Supplementary Fig. S3), suggesting that some DI-290 RNA may be secreted by HEK-DI-290 cells in exosomes. The source of pelleted DI-290 RNA from HEK-DI-290 cells not captured by anti-CD63 antibody Dynabeads is unknown.

### Infectious DENV-2 was not detected in supernatant from cultures producing DIPs

The HEK-DI-290-ORF cells contained codon optimized DENV-2 genomic DNA split into two pieces that, separately, encoded S and NS proteins. To exclude the possibility that HEK-DI-290-ORF cells might produce infectious DENV-2 RNA, supernatant from HEK-DI-290-ORF cultures were added to Vero cells and 7 days later, the cell lysate was analyzed by western blot for the presence of DENV-2 NS protein. NS3 was only detected in lysate of Vero cells infected with 0.01 plaque forming units (p.f.u.) of DENV 2 (Fig. 3c), but not from Vero cells incubated with HEK-DI-290-ORF culture supernatant, indicating that HEK-DI-290-ORF cells do not produce infectious DENV-2.

HEK-DI-290-ORF cells were grown in a glass spinner flask using serum free medium to enable continuous DIP production. HEK-DI-290-ORF cells cultured this way produced DIP supernatants that contained up to 9 × 10^6^ DI-290 RNA copies/mL (Fig. 3d) (Supplementary Data 1). We performed a plaque assay to determine if infectious dengue virus was present in DIP supernatant. Vero cells were incubated with culture supernatant containing DENV-2 (equivalent to 3 × 10^7^ copies of NS5) or with concentrated DIP (containing 3 × 10^7^ copies of DI-290 RNA) (Fig. 3e). After 5 days, immuno-plaque analysis showed that DENV-2 titer was ~1.2 × 10^5^ p.f.u./mL (Fig. 3f) (Supplementary Data 1). No plaques were detected in Vero cells incubated with DIPs indicating that infectious DENV virus was not present in the DIP supernatant (Fig. 3e,f).

### The sedimentation rate of DIPs is reduced compared to DENV-2 on a velocity gradient

**Fig. 4:**
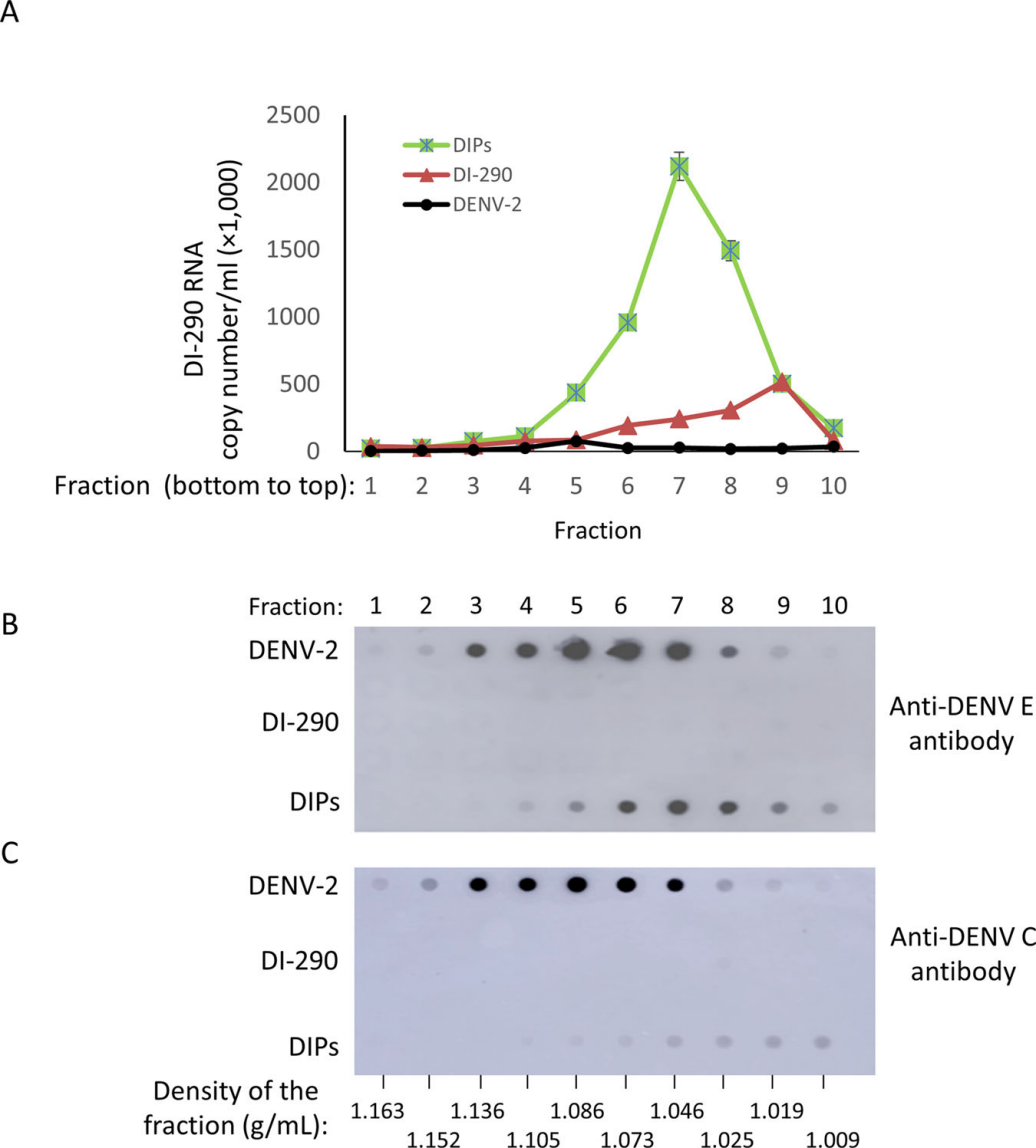
Velocity gradient analysis of DIPs compared to DENV-2. DIPs purified from HEK-DI-290-ORF cell culture supernatant, purified DENV-2 from infected HEK 293T cells or CHT column purified supernatant from HEK-DI-290 were placed on a 5–50% sucrose gradient and subjected to ultracentrifugation. Gradient fractions were collected from the bottom of the tube and **A** RNA was extracted from triplicate samples of each fraction that was assayed by RT-qPCR to measure levels of DI-290 RNA. The mean values and SD are shown. Each fraction was assayed by immuno-dot-blot using **B** anti-DENV-2 E or **C** anti-DENV-2 C antibodies. A representative of two experiments with similar results is shown.

DIPs in culture supernatant were applied to a 5–50% w/v sucrose velocity gradient and centrifuged for 3 h. Gradient fractions were collected and analyzed for DI-290 RNA copy numbers (Fig. 4a) (Supplementary Data 1), and for levels of DENV2 E and C proteins by immuno-dot blot (Fig. 4b, c). Supernatant from cultures of HEK-DI-290 cells (i.e., DI-290 RNA only) and DENV-2 from infected HEK 293T cells were used as a negative control and a positive control, respectively. The highest levels of DI-290 RNA were observed in gradient fractions 6–8 (Fig. 4a), where high levels of E also were observed (Fig. 4b). The C protein also was found in these fractions although the highest levels were observed in fractions 7–10. The density of fractions 6–8 ranged from 1.073 to 1.025 g/mL, respectively. The highest levels of DENV-2 E were seen in fractions 5–7 where the density ranged from 1.086 to 1.046 g/mL (Fig. 4c, b). The levels of DI-290 RNA from the negative control supernatant sample peaked in fraction 9, and were ~approximately five-fold lower that the DIP DI-290 RNA peak. In DENV-2 samples, DI-290 RNA was detected but at a >50-fold lower level in fraction 5 compared DIP sample in fraction 7 (Fig. 4a). Sedimentation analysis shows that HEK-DI-290-ORF cells produce DIPs associated with DI-290 RNA that in a sucrose gradient have a reduced sedimentation rate compared to DENV-2.

### HEK-DI-290-ORF cells produce DIPs that transmit DI-290 RNA to target cells expressing DENV S and NS proteins where it is replicated

**Fig. 5:**
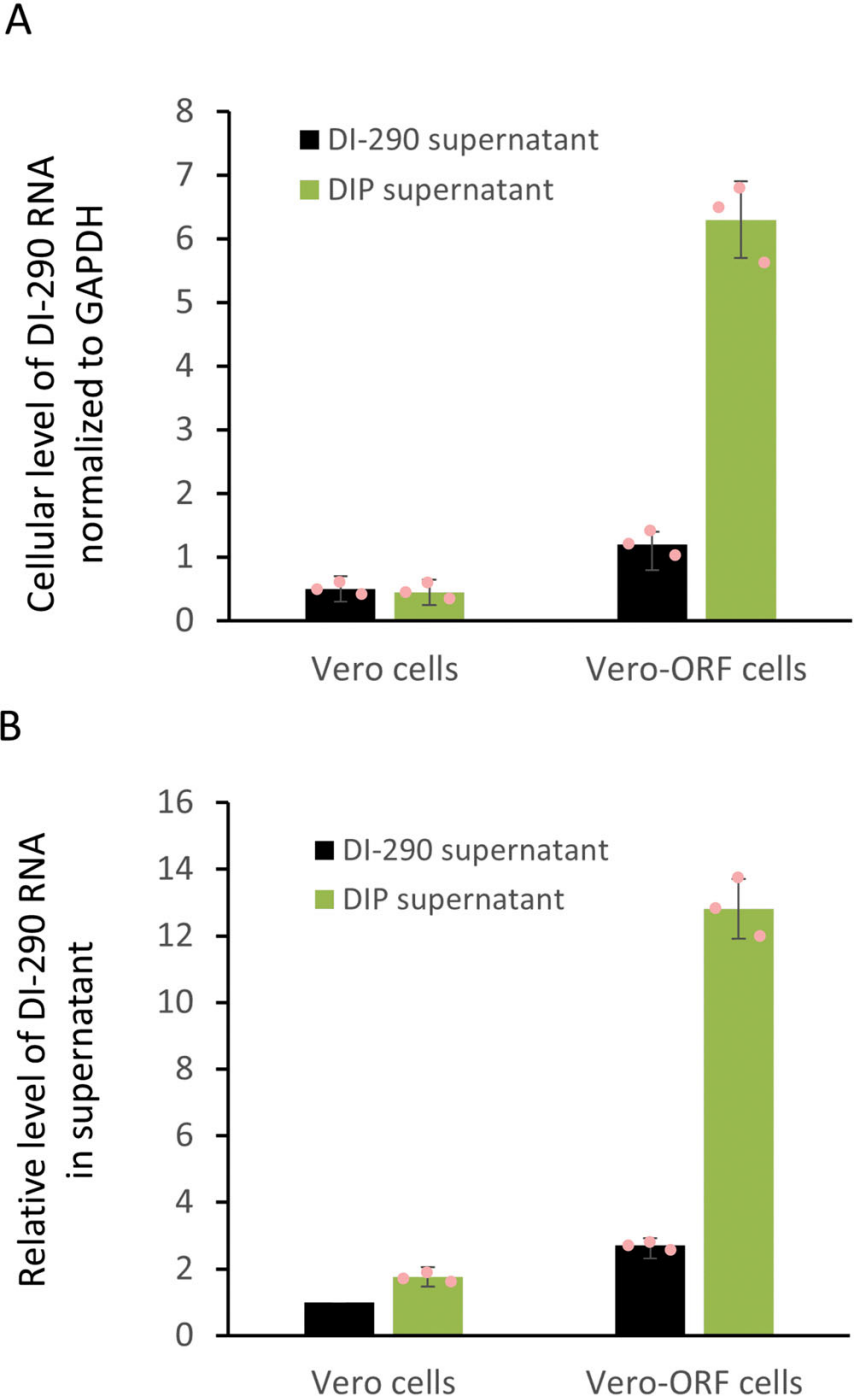
DIPs and their DI-290 RNA are a transmissible VLP. Vero cells or Vero-ORF expressing DENV-2 S and NS proteins were treated with cell-free culture supernatant of HEK-DI-290 (black bars, a negative control) or DIP supernatant of HEK-DI-290-ORF cells (red bars) for 12 h when the culture medium was replaced. After 48 h, RNA samples were prepared from **A** cells and **B** culture supernatant and used in RT-qPCR assays to measure levels of DI-290 RNA. The cellular levels of DI-290 were normalized to GAPDH mRNA in the same sample. The relative level of DI-290 in Vero cells treated with negative control supernatant was set as 1. The error bars indicate the SD. A representative of three experiments with similar results is shown. .

DIPs are intended to deliver DI-290 RNA to a DENV-infected cells where the DI RNA replicates, virus replication is inhibited and new DIPs are produced. To test for DI RNA delivery, replication and new DIP production in target cells, a Vero-ORF cell line that expressed the DENV-2 S and NS proteins (Supplementary Fig. S1), but not DI-290 RNA, was used. Vero-ORF cells allow for measurement of DI-290 replication and the production of new DIPs. Vero-ORF cells were incubated with supernatant from cultures of HEK-DI-290-ORF (DIPs) or HEK-DI-290 (DI-290 control supernatant) cells that contained equivalent amounts of DI-290 RNA. The inoculum was replaced with new culture medium after 12 h and incubated for an additional 48 h at 37 °C. The Vero-ORF cells (Fig. 5a) and culture supernatant (Fig. 5b) were collected and total RNA was extracted from both fractions followed by RT-qPCR to measure DI-290 RNA levels. The levels of cellular GAPDH mRNA also was measured and used to normalize levels of DI-290 RNA in cellular total RNA samples. Incubation of DIP supernatant with Vero-ORF cells produced cellular levels of DI-290 RNA that was ~600% higher compared to samples from Vero cells incubated with either DIP supernatant or the DI-290 control supernatant. Similarly, Vero-ORF cells incubated with DIPs had DI-290 RNA levels that were 700–1200% higher than Vero cells incubated with DIPs or the DI-290 control supernatant negative. Interestingly, Vero-ORF cells incubated with DI-290 supernatant consistently had about twofold higher amounts of DI-290 RNA in culture supernatant compared to Vero cells (Fig. 5a, b) (Supplementary Data 1). While not formally investigated, it is possible that extracellular vesicles such as an exosome containing DI-290 RNA (Supplementary Fig. S3) explain higher amounts of DI RNA observed in Vero-ORF cells incubated with DI-290 supernatant, i.e., via exosomal delivery of an RNA cargo^30^. These results showed that DIPs produced by HEK-DI-290-ORF cells could transmit DI-290 RNA to a target cell and that the DI-290 RNA could be replicated if the target cell expressed DENV-2 S and NS proteins.

### DIPs inhibited DENV-2 replication in Huh7 and Vero cells

**Fig. 6:**
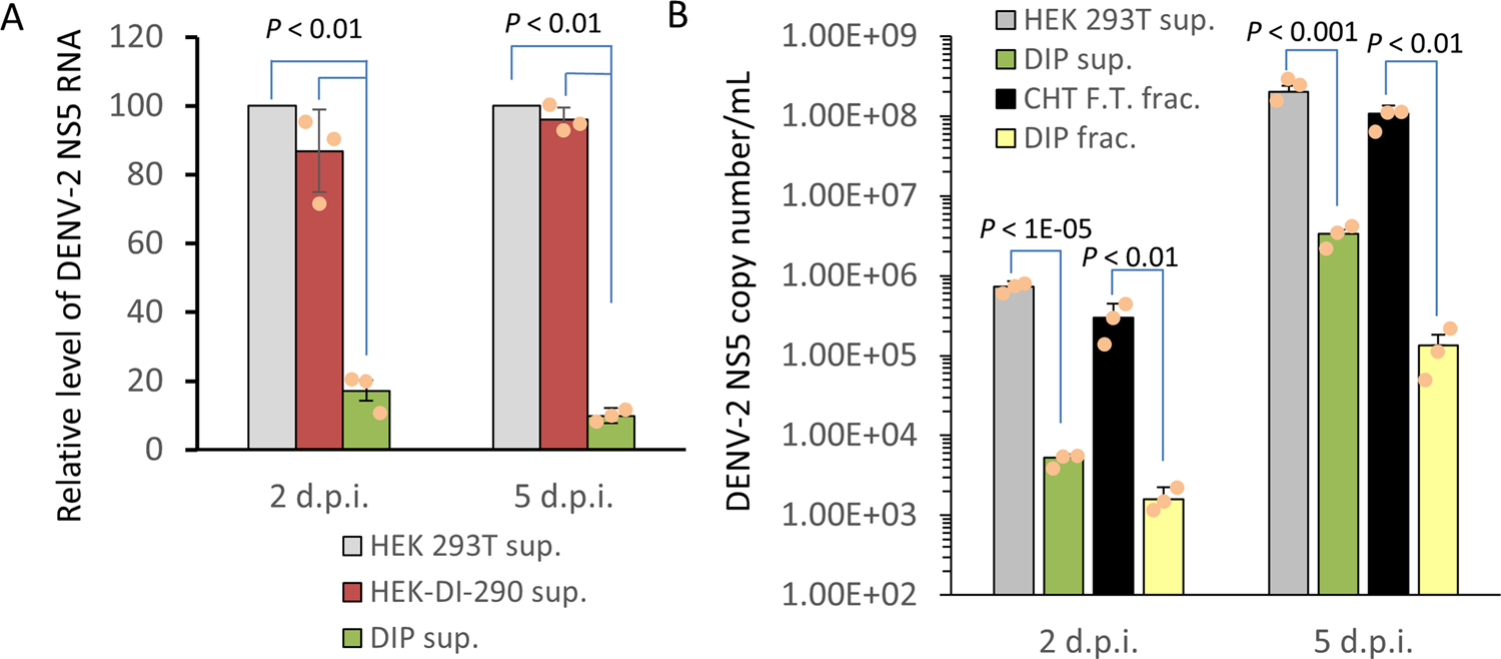
Unpurified DIPs and CHT column purified DIPs can inhibit DENV-2 replication in Huh7 cells. **A** Huh7 cells in triplicate wells were infected with DENV-2 at an MOI of 1 for 3 h. The virus was removed and the cells were treated with either cell-free supernatant from HEK 293T cells, HEK-DI-290 cells or DIPs from HEK-DI-290-ORF cells. For the latter two treatments, DI-290 RNA was used at five copies of DI-290 RNA/cell. Culture supernatant from each well was collected at the time points indicated (d.p.i.), and the level of DENV-2 RNA in each sample was measured by RT-qPCR using primers to detect the NS5 region. Infection in the presence of HEK 293T supernatant was set at 100%. Error bars indicate the SD. A *P* value comparing negative control treatments to DIPs is shown. **B** As in **A**, DENV-2 infected Huh7 cells were treated with The DIP fraction (DIP frac.), DIP in culture supernatant (DIP sup), the CHT flow-through fraction (CHT F.T. frac.) or HEK 293T supernatant (HEK 293T sup.) as a negative control. DI-290 RNA levels for the first three treatments were normalized to ten copies of DI-290 RNA/cell. At the time points indicated, the level of DENV-2 RNA in culture supernatant was measured by RT-qPCR using primers for the DENV-2 NS5 region. The *P* values shown were calculated with a two-tailed Student’s *t* test. A representative of three experiments with similar results is shown.

Huh7 cells were infected with DENV-2 at a multiplicity of infection (MOI) of 1 for 3 h. The inoculum was replaced with cell-free culture supernatant from HEK-DI-290-ORF cells (DIP supernatant), from HEK-DI-290 cells that produce DI-290 RNA but cannot make DIPs (see Fig. 3a), or from HEK 293T cells that do not make DI-290 RNA or DENV proteins. The first two supernatants each had a DI-290 RNA concentration equivalent to 5 copies per cell. The two later supernatants lack DIPs and were added as negative controls. All treatments were replaced with new growth medium after 15 h. At 2 and 5 days post-infection (d.p.i.), DENV-2 RNA present in culture supernatant was quantified (Fig. 6a) (Supplementary Data 1). There were five to tenfold lower levels of DENV-2 RNA in culture supernatant when the DENV-2 infected cells were incubated with DIP supernatant compared to control supernatants. Similar results were obtained when these experiments were performed using Vero E6 in place of Huh7 cells (Supplementary Fig. S4). The results indicate that DI-290 RNA conveyed by DIPs imparted a strong anti-DENV effect; a feature that has been observed for naturally occurring DIPs^9^.

### Ceramic hydroxyapatite (CHT) column purified DIPs inhibit DENV-2

The DIPs in culture supernatant of HEK-DI-290-ORF cells were purified using CHT column chromatography as was previously described to purify infectious DENV^31^. Analysis of the eluted DIP fraction showed an ~4.6-fold increase in DI-290 RNA concentration compared to the original HEK-DI-290-ORF culture supernatant (Supplementary Fig. S5). The concentration of DI-290 RNA detected in the flow-through (F.T.) fraction was 46% of the original DIP culture supernatant. The nature of the DI-290 in the F.T fraction is unknown. Possibilities include that the DIP binding capacity of the CHT column may have been exceeded, or the DI-290 RNA in the F.T. fraction may not bind to CHT.

The antiviral activity of the CHT purified DIP fraction was compared to the original DIP culture supernatant (i.e., unpurified DIPs) and the CHT F.T. fraction. Huh7 cells were infected with DENV-2 at an m.o.i of 1 for 3 h. The virus inoculum was removed and the infected cells were treated with culture medium spiked with the original DIP supernatant, the F.T. fraction or the DIP fraction, where each treatment contained a DI-290 RNA concentration equivalent to ten copies of DI-290 RNA per cell. DENV-2 infected Huh7 cells were incubated with culture supernatant for HEK 293T cells as a negative control. The treatments were removed from the cells after 15 h and replaced with new culture medium. Samples of culture supernatant were collected at 2 and 5 d.p.i. and assayed for levels of viral RNA by RT-qPCR using oligonucleotide primers to DENV-2 NS5 gene. Both DIP supernatant and CHT-purified DIPs inhibited DENV-2 replication by >100-fold at both time points (Fig. 6b) (Supplementary Data 1). DENV-2 replication was not inhibited significantly when the F.T fraction was added suggesting that the DI-290 RNA in the F.T may not be associated with functional DIPs. The assays indicates that DIPs purified by CHT chromatography retain anti-DENV-2 activity.

### Purified DIPs inhibit DENV-2 in a dose-dependent manner and are a pan-serotype DENV inhibitor

**Fig. 7:**
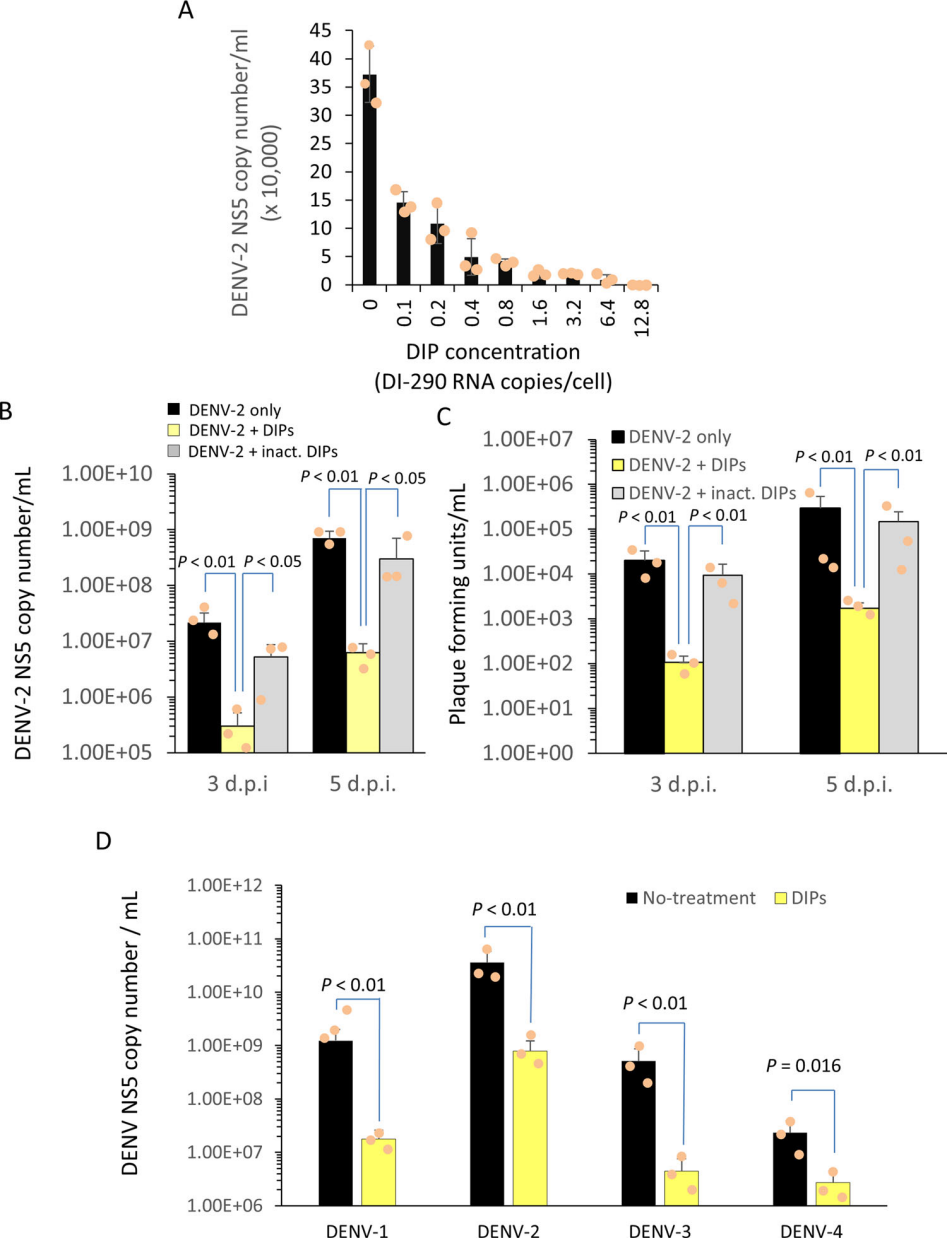
DIPs inhibit DENV-2 in a dose-dependent manner and are a pan-serotype DENV inhibitor. **A** Triplicate wells of DENV-2 infected HuH7 cells (MOI 1.0 for 3 h) were treated with serially diluted DIPs as shown. After 2 d.p.i., the level of viral RNA in culture supernatant was measures by RT-qPCR using primers to the DENV-2 NS5 region. Mean values are shown with error bars indicating the SD. Using triplicate wells, DENV-2 replication in infected cells (DENV-2 only) was compared to cells treated with purified DIPs (DENV-2 + inact. DIPs) or with DIPs treated with UV light irradiation (DENV-2 + inact. DIPs). In **B**, the level of viral RNA in culture supernatant was measure by RT-qPCR as previously described. In **C**, viral titers in culture supernatant were measure using an immuno-plaque assay. **B**, **C** Mean values are shown and error bars show the SD. The *P* values shown were calculated with a two-tailed Student’s *t* test. **D** In triplicate wells, Huh7 cells were infected with prototype strains of all DENV serotypes at an MOI of 1 for 3 h. The infected cells were treated with purified DIPs equivalent to five copies of DI-290 RNA/cell. The levels of DENV-2 RNA in culture supernatant were measured by RT-qPCR as previously described. All measurements show mean values and error bars indicate the SD. The *P* values shown were calculated with a two-tailed Student’s *t* test. The data show representative outcome of two identical experiments with similar results.

The buffer in the DIP fraction was exchanged with 1 × PBS and the DIPs were concentrated by centrifugal filtration using a 100 kDa cutoff membrane. Huh7 cells were infected with DENV-2 as described above and then treated with serially diluted DIPs as indicated (Fig. 7a) (Supplementary Data 1). The experiments showed dose-dependent inhibition of DENV-2 replication by DIPs in Huh7 cells.

Germicidal UV-irradiation can inactivate an RNA virus by inducing photodimeric lesions in the genome^32,33^. Huh7 cells were infected with DENV-2 as before and then treated with DIPs equivalent to a DI-290 RNA concentration of 10 copies per cell or with the same amount of DIPs inactivated by germicidal UV-irradiation. Analysis of Huh7 culture supernatant sampled at 3 and 5 d.p.i. showed that DIP treatment of infected cells reduced DENV-2 genomic RNA levels by Log_10_ 1.89 and Log_10_ 2.07 copies /mL, respectively (Fig. 6a). In the same samples, DIPs reduced levels of infectious virus by Log_10_ 2.22 and Log_10_ 2.14, respectively (Fig. 7b) (Supplementary Data 1). UV-irradiation of DIPs reduced inhibition of DENV genomic RNA levels and infectious virus titers by >99% (Fig. 7b,c) (Supplementary Data 1). The outcomes provides further evidence that DI-290 RNA inhibits DENV-2 replication in Huh7 cells.

Huh7 cells were infected at an MOI of 1 for 3 h using DENV prototype strains representing the four serotypes. After removing the virus inoculum, purified DIPs equivalent to five copies of DI-290 RNA per cell were added to each culture and incubated for 15 h when the medium was replaced. Cell culture supernatant was collected at 3 d.p.i. and the level of virus replication was examined by RT-qPCR using NS5 primers specific for each DENV serotype (Fig. 7d) (Supplementary Data 1). The results showed that DIPs inhibited replication of DENV serotypes 1, 2, and 3 by >97% and subtype 4 by 88% in Huh7 cells. DIPs conveying DI-290 RNA are a pan-serotype DENV inhibitor.

## Discussion

This study showed that DIPs conveying a 290 nt long DI RNA, i.e., DI-290 RNA, derived from DENV-2 have a broad acting, pan-serotype anti-DENV activity. The DIP production system developed here can continuously produce DENV-2 derived DIPs in vitro that can be purified from culture supernatant and concentrated, which retain anti-DENV activity. Moreover, DIPs made by this system are free of infectious DENV-2. This is important because DIPs have been long recognized a potential antiviral therapeutic agent, which has been has been inferred by in vivo studies where loss in viral pathogenicity and improvements in recovery rates from viral infection in animal hosts due to DIPs has been reported^18,19^. The therapeutic potential of DIPs generated from IAV, vesicular stomatitis virus, Semliki Forest virus, and other viruses, to reduce viral pathology in animals reviewed in ^22^, and to reduce virus replication in cell-based assays for other human pathogenic viruses such as DENV^9,10,34^ and Nipah (NiV)^35^, supports the possibility that DIPs therapy could be a viable antiviral strategy. However, production of DIPs from viruses, such as IAV and NiV, typically requires WT “helper” viruses, which can be difficult to inactivate or to remove^22,35,36^. Recently, a system using a combination of HEK 293T and MDCK cell lines stably expressing the IAV PB2 protein and transiently expressing the other IAV gene segments using plasmids was used to produce infectious virus-free IAV DIPs^21^. The DENV DIP production system described in this study differ from the IAV system because DIP synthesis by HEK-DI-290-ORF cells is continuous, infectious virus-free and potentially scalable, which will enable further preclinical studies in animal models of DENV infection.

The studies by Gard, von Magnus and others reported a reduced rate of sedimentation of “incomplete” viruses associated with “autointerference” of IAV infectivity^37^, presumably caused by IAV DIPs having reduced amounts of viral genomic RNA compared to infectious virus^22^. We observed that DENV-2 DIPs had a reduced rate of sedimentation compared to WT virus (Fig. 4a,b), which is most likely due to the small size of DI-290 RNA that is only ~2.5% of the full length DENV-2 viral genome. However we cannot exclude other possibilities such as that DIP protein content may be a factor affecting sedimentation in a sucrose velocity gradient.

We confirmed that DIPs had antiviral activity against DENV-2 replication in Huh7 cells (Fig. 6) and Vero cells (Supplementary Fig. S5). The mechanisms of antiviral activity by DIPs and required factors are not completely understood, but interference of WT virus replication by DI RNA competing for viral and cellular resource as well as stimulation of host immunity are likely involved^38–40^. Vero cells lack most type-1 interferon responses, so DI-290 RNA antiviral activity in Vero cells suggests that DI-290 inhibits DENV replication by multiple mechanisms including direct competition for cellular or viral resources in cells. We purified DIPs by a method previously used to purify infectious IAV and DENV-2^31^, where virion particles binds to CHT and are eluted with a sodium phosphate buffer (Fig. 6b). The CHT purified DIPs retained antiviral activity (Fig. 6b), and inhibited DENV-2 in a dose-dependent manner (Fig. 7A). DENV-2 genomic RNA replication and infectious virus production were inhibited by DIP treatment (Fig. 7b, c). DIP antiviral activity could be inactivated by germicidal UV irradiation suggesting that intact DI-290 RNA was required for inhibition^32^. When cells were infected by DENV at an MOI of 1, modest but significant inhibition of DENV-2 replication was observed by treating cells with as little as 0.1 copy of DI-290 RNA per cell (Fig. 7a). This result suggests that inhibition of DENV-2 by DIPs required DI RNA replication and cell to cell transmission of DIPs. Cells treated with ~12 copies per cells inhibited DENV-2 replication by 99.4%. In comparison, IAV and Nipah virus (NiV)-based DIPs are reported to require from ~1000 to ~5000:1 ratios of DI RNA to viral genome to inhibit infectious virus replication by greater than 99% (for IAV) and 99.9% (for NiV)^21,35^. A speculation is that short DI genomes are good inhibitors because they “out replicate” the viral genome in a competitive manner. A natural IAV-derived anti-IAV DIP has a DI RNA called DI-244 that contains only 395 of the 2341 nt of IAV genome segment 1. Investigations of NiV DI RNA showed that, overall, relatively short, naturally derived copyback DI genomes see^35^ from 378 nt to 1470 nt (of a 18,246 nt genome) had good antiviral activity. Just as DENV intact genomic RNA, DI-290 RNA has the RNA sequences and elements in the 5′UTR (5′SLA, 5′SLB, cHP, and the 5’CS) and a partial 3′ UTR region (CS1, sHP, and 3′SL) that are highly conserved by DENV and required DENV genomic RNA replication^9,41–43^. Overall, the evidence suggests that DI-290 RNA interacts with important viral^44^, or cellular proteins required for DENV replication and packaging^45,46^.

A broad antiviral activity was also reported from IAV DIPs that inhibited replication of IAV, influenza B virus and pneumonia virus in mice^47,48^. The DIPs produced from our in vitro system are DENV-2 based, but strongly inhibited all dengue serotype viruses in Huh7 cells (Fig. 7d). This result suggests that DI-290 RNA can replicate with the help of dengue serotype 1, 3, and 4 viruses, which is consistent to our early results that subgenomic RNAs derived from dengue serotype 1, 3, and 4 viruses can replicate in DENV-2 infected cells, and the RNA structures, rather than the RNA sequences are more important for DENVs replication^10^. We cautiously speculate that antibody-dependent enhancement, by which heterotypic antibodies of a previously infected DENV patient bind but do not neutralize virions of a subsequent infecting DENV type to enhance virus replication and exacerbate illness^49,50^, in patients could possibly help the broad antiviral activity of DIPs. This possibility will require future testing in an appropriate animal model.

In summary, we created a novel DIP production system and demonstrated that DIPs are robust pan-subtype inhibitor of DENV in cell based assays. The system can up-scaled and DIPs can be purified and concentrated, which makes a future clinical application of anti-DENV DIPs a possibility. Although we have achieved proof-of-principal that DENV DIPs ability to inhibit DENV replication in vitro, the project should progress to animal models of DENV infection to determine if DIPs can reduce viral burden in vivo. DIPs have been identified from many RNA viruses including coronaviruses^22,40^. Our successful in vitro DIPs production system is an approach that could be used to develop novel inhibitors for other viral pathogens, such as SARS-CoV-2, that lack a specific antiviral.

## Methods

### Plasmid constructs

The lentiviral vector pCDH-EF1α-MCS-BGH-PGK-GFP-T2A-Puro was a gift from Stacey Edward, QIMR Berghofer Medical Research Institute, Australia and pCMV∆R8.91 was a gift from Andreas Suhrbier, QIMR Berghofer Medical Research Institute, Australia. pCMV-VSV-G was obtained from Ian Mackay, the University of Queensland, Australia. The pCFP-coilin plasmid was a gift from Miroslav Dundr from Rosalind Franklin University, USA. The pcDNA3.MLV.GP (MLV Gag-Pol) and pSERS11-SF-EGFP-PRE were gifts from Axel Schambach, Hannover Medical School, Germany. pSERS11-EF1α-mCherry-T2A-PRE expression plasmid was generated by inverse PCR of EF1α-mCherry from pSicoR-EF1α-mCh (obtained from Addgene, plasmid #31847), which were then inserted into the pSERS11-SF-EGFP-PRE via Xba I and Not I restriction sites.

A human codon optimized DENV-2 structural gene encoding CprME and an African green monkey codon optimized DENV-2 non-structural gene encoding NS1-NS5 DNA sequences were synthesized by GenScript (USA) based on the DENV-2 infectious clone pWSK602 sequence^9^. The codon optimized sequences were then amplified by PCR using CloneAmp HiFi premix polymerase (Clontech). The PCR products were then inserted into the vectors pSERS11-EF1α-mCherry-T2A-PRE and pCDH-EF1α-GFP-T2A-Puro respectively by an in-fusion reaction (Clontech) to make the plasmids pSERS11-EF1α-mCherry-T2A-DENV-2-CprME and pCDH-EF1α-DENV-2-NS1-NS5-BGH-PGK-GFP-T2A-Puro. To create the pCDH-EF1α -MCS-BGH-PGK-CFP-T2A-Puro plasmid, the CFP was PCR amplified from pCFP-coilin, which was then inserted into the pCDH-EF1-MCS-BGH-PGK- GFP-T2A-Puro using In-Fusion cloning kit according to the manufacturers’ instructions. pCDH-CMV-DI-290-HDVr-BGH-PGK-CFP-T2A-Puro was generated by inserting the synthetically made DNA CMV-DI 290-HDVr (GenScript) into the pCDH-EF1α -MCS-BGH-PGK-CFP-T2A-Puro via Hpa I and Not I restriction sites. The sequence of DI-290 subgenomic RNA can be found in GenBank (Accession: HM016526.1 GI: 295702064).

### Cell lines and virus-like particle (VLP) production

HEK293T, Vero E6 (Vero), Huh7, and Phoenix-Ampho cells were cultured in Dulbecco’s Modified Eagle medium (DMEM) (Life Technologies) supplemented with 10% (v/v) fetal bovine serum (FBS) (Life Technologies) and 1% (v/v) penicillin–streptomycin. All cells were incubated at 37 °C in a humidified 5% CO_2_ atmosphere.

To generate lentiviral particles containing the DENV-2 NS1-NS5 ORF or CMV-DI 290-HDVr cassette gene, HEK293T cells were co-transfected with pCMV∆R8.91, pCMV-VSV-G and pCDH-EF1-NS1-NS5-BGH-PGK-GFP-T2A-Puroor pCDH-CMV-DI 290-HDVr-BGH-PGK-CFP-T2A-Puro using X-tremeGENE HP DNA transfection reagent (Roche) according to the manufacturers’ instructions. VLPs containing DENV-2 CprME gene were produced in Phoenix-amphotropic retroviral packaging producer cell line by co-transfection of pSERS11-EF1α-mCherry-T2A-CprME and pcDNA3.MLV.GP (MLV Gag-Pol expressing plasmid) using X-tremeGENE HP DNA transfection reagent according to the manufacturers’ instructions. At 48 h post-transfection, cell culture supernatants containing VLPs were collected, filtered through a 0.45 μm filter and stored in small aliquots at −80 °C until needed.

### Generation of cell lines stably expressing DENV-2 viral proteins and DENV-2 DI-290 RNA producing DIPs

HEK 293T or Vero cells were transduced with VLPs as described above: HEK 293T transduced with the lentiviral VLPs containing DI-290 gene, to make the HEK-DI-290 cell line; Vero cells transduced with the lentiviral VLPs, containing DENV-2-NS1-NS5 gene, and retroviral VLPs, containing DENV-2-CprME gene, to obtain the Vero-ORF cell line; and HEK 293T cells transduced with VLPs containing DI-290, DENV-2-NS1-NS5 and DENV-2-CprME genes to create the HEK-DI-290-ORF cell line. At 72 h post-transduction, the cells were trypsinized, filtered through 37 μm Nylon Mesh to remove cellular clumps and sorted with a FACS ARIA III cell sorter (BD Biosciences).

HEK-DI-290-ORF cells were adapted to grow in glass spinner flask (Corning Inc., USA) in Ex-Cell 293 serum-free medium (Merck) using a magnetic stir plate (Thermo Fischer Scientific) set at 100 RPM, which were incubated at 37 °C in a humidified 5% CO_2_ atmosphere. HEK-DI-290-ORF cell were seeded in at 2 × 10^5^ cells/mL and grown for 48 h when the supernatant was collected and filtered through a 0.45 µm membrane. DIPs were concentrated using an Amicon Ultra-15 Centrifugal filter with a 100 kDa cutoff (Merck) by centrifugation at 4000 × *g* for 25 min at 4 °C.

### Immunofluorescence analysis

Cells were grown on glass coverslips, fixed in 4% (w/v) paraformaldehyde at room temperature for 10 min and quenched with 50 mM NH_4_Cl for 5 min. Cells were then permeabilized with 0.1% (v/v) Triton X-100 for 15 min and blocked in 10% (v/v) normal goat serum (Sigma Aldrich) for 15 min. DENV-2 NS3 protein was detected with a rabbit anti-DENV NS3 polyclonal antibody (Sigma Aldrich). DENV-2 E and C were probed with a rabbit anti-DENV E polyclonal antibody (GeneTex) and a rabbit anti-DENV C polyclonal antibody (Novusbio), respectively. Double strand (ds) RNA was probed with a mouse anti-dsRNA monoclonal antibody J2 (SCICONS). The secondary antibodies were Alexa Fluor 647-conjugated goat anti-rabbit (Thermo Fisher Scientific) or Cy5-conjugated goat anti-mouse (Life Technologies). Nuclei were stained with 1 µM 4′,6-diamidino-2-phenylindole (DAPI) (Life Technologies). The coverslips were mounted onto slides with ProLong Gold antifade reagent (Life Technologies). Fluorescent images were captured using a Zeiss 780 NLO confocal scanning microscope (Zeiss) with 63× objective lenses and standard lasers and filters for Alexa Fluor 647, Cy5, and DAPI fluorescence.

### Western blot analysis

Cell were lysed at 4 °C for 30 min with lysis buffer (50 mM Tris-HCl, pH 7.4; 150 mM NaCl; 1 mM EDTA; 1% [v/v] Triton X-100; protease inhibitor cocktail [Roche]). Cell lysates were centrifuged at 12,000 × *g* for 10 min and clarified supernatants were collected. The total protein concentrations were determined by the Bradford method against a bovine serum albumin standard. Protein was boiled in SDS-PAGE sample buffer (125 mM Tris–HCl, pH 6.8; 4% [v/v] SDS; 20% [v/v] glycerol; 0.004% [w/v] bromphenol blue), and separated by 10% SDS-PAGE. Gels were electro-blotted onto a polyvinylidene fluoride membrane (Pall) using a semi-dry transfer system (Bio-Rad). DENV-2 E and CA proteins were detected with a rabbit anti-DENV E polyclonal antibody (GeneTex) and a rabbit anti-DENV C polyclonal antibody (Novusbio), respectively. DENV-2 NS3 and DENV-2 NS5 were detected with a rabbit anti-DENV NS3 polyclonal antibody (Sigma Aldrich) and a mouse anti-DENV NS5 monoclonal antibody (GeneTex). dsRNA was detected with a mouse anti-dsRNA monoclonal antibody J2 (SCICONS). Endogenous β-tubulin was detected with a mouse anti-β-tubulin monoclonal antibody (Sigma Aldrich). Primary antibodies were detected with anti-rabbit IgG horseradish peroxidase (HRP)-linked antibodies or anti-mouse IgG HRP-linked antibodies (Cell Signaling Technology).

### RNA extraction and RT-qPCR assay

Total RNA from cells was isolated with TRIzol reagent (Thermo Fisher Scientific) accordance with the manufacturer’s protocol. For culture supernatant RNA extraction, supernatants were treated with Benzonase nuclease (Merck) at 20 U/mL for 30 min at room temperature, centrifuged at 1000 × *g* for 10 min and passed through a 0.45 µm filter. Then, the clarified supernatants were pelleted by ultra-centrifugation at 100,000 × *g* for 1 h at 4 °C. The total RNA from the pellet was isolated with TRIzol reagent. cDNA was made using random hexamer primers (New England Biolabs) and Superscript IV reverse transcriptase (Thermo Fisher Scientific) according to the manufacturers’ instructions. DI-290 RNA, DENV-2 E, NS1, and NS5 RNA regions and GAPDH RNA was quantified by qPCR using specific primers. The primer sequences are available on request.

### Velocity gradient analysis

Culture supernatants (1 mL) from HEK-DI-290, HEK-DI-290-ORF or supernatant from DENV-2 infected HEK 293T cells were each layered on top of 10 mL 5–50% w/v sucrose/PBS (pH 7.4) gradient and centrifuged at 80,000 × *g* for 3 h at 4 °C in a SW40i rotor (Beckman). Fractions (1 mL) were collected from the gradient by puncturing the bottom of the centrifuge tube. RNA from 100 µl of fractionated samples was extracted and the levels of DI-290 RNA were assayed by RT-qPCR using oligonucleotide primers for the DENV-2 NS5 gene.

### Immuno-dot blotting

Fractions from each sucrose gradient (200 µl) were applied to a nitrocellulose membrane (Amersham Biosciences) with a dot blot apparatus. The membrane was blocked in 5% w/v skim milk in PBST for 1 h at room temperature before being incubated in rabbit anti-DENV E polyclonal antibodies (GeneTex) and rabbit anti-DENV C polyclonal antibodies (Novusbio) overnight at 4 °C. Primary antibodies were detected with anti-rabbit IgG horseradish peroxidase (HRP)-linked antibodies or anti-mouse IgG HRP-linked antibodies (Cell Signaling Technology).

### Immuno-plaque assay

Vero cells were seeded in a 24-well plate (2 × 10^5^ cells/well) and incubated overnight to produce a confluent monolayer. The culture medium was removed the next day and the cells were washed once with 1 × PBS. Then, a 200 µL aliquot of ten-fold serially diluted DENV-2 or DENV DIP in DMEM supplemented with 10% (v/v) FBS was added to each well in triplicate and incubated for 2 h at 37 °C. The plates were rocked every 20 min and then 800 µl of 1% (W/V) high viscosity carboxymethyl cellulose overlay (Sigma Aldrich) in Medium 199 (Sigma Aldrich) containing 2% FBS was added. After 5 days of incubation, the cells were washed with PBS and fixed with cold (−20 °C) 1:1 (v/v) acetone/methanol for 5 min at room temperature. The fixative was removed and the cells were air dried for 1 h at room temperature. The cells were blocked with Odyssey blocking buffer (LI-COR Biotechnology) for 1 h at 37 °C and probed with mouse anti-Flavivirus E antibodies 4G2 (produced from culture supernatant of hybridoma D1-4G2-4-15) for 1 h at 37 °C, followed by detection with IRDye 800CW anti-mouse IgG (LI-COR Biotechnology) using LI-COR Odyssey CLx infrared imaging system (LI-COR Biotechnology).

### CHT ceramic hydroxyapatite chromatography and concentration of DIPs

CHT has been used previously to purify DENV and IAV^31^. A column (15 mm × 100 mm, Bio-Rad Laboratories) was packed with 1 mL of 40 µm CHT™ ceramic hydroxyapatite Type II Media (Bio-Rad Laboratories) and set on a L/S MFLEX Easy-Load system (Cole-Parmer Instrument Co.) at a flow rate was 1 mL/min. The column was washed with 600 mM sodium phosphate buffer (NaPB) pH 7.2 and then equilibrated with 10 mM NaPB pH 7.2. 1. Then 50 mL of clarified culture supernatant that was filtered through a 0.45 µM membrane was then loaded onto the column. The column was washed with 20 mL of 10 mM NaPB pH 7.2, which was collected and is the F.T. fraction. The column was eluted with 5 mL of 250 mM NaPB pH 7.2, which is the DIP fraction. The DIP fraction and 5 mL of the F.T. fraction were washed and concentrated to 500 µL in 1× PBS using an Amicon Ultra Centrifugal Filter with a 100 kDa cut-off (Merck) by centrifugation at 4000 × *g* for 25 min at 4 °C. The DIP concentrate was stored in small aliquots at −80 °C until needed.

### Analysis of DENV DIP antiviral activity

Vero or Huh7 cells were seeded in 12-well plates at a density of 100,000 cells/well. The next day, the cells were infected with DENV-1, -2, -3, or -4 at MOIs of 0.01 or 1 depending on the experiment. The DENVs subtypes had been isolated from dengue patients as described in a previous publication^9^. At 3 h post-infection, the cells were washed once with PBS and incubated in 1 mL of culture medium containing DIPs at the concentration indicated in the results section. After 15 h of DIP treatment, the medium was replaced. At 2, 3, or 5 d.p.i, (as indicated in the experiment) 100 μL of culture supernatant was collected and the concentration of DENV-2 genomic RNA in the sample was measured by RT-qPCR using primers to DENV-2 NS5 gene.

### Statistics and reproducibility

Statistical analysis was performed with a two-tailed Student’s *t* test from at least three independent experiments or measurements. Statistical significance was set at *P* < 0.05.

### Reporting summary

Further information on research design is available in the Nature Research Reporting Summary linked to this article.

## Supplementary information

Supplementary Information

Description of Additional Supplementary Files

Supplementary Data 1

Reporting Summary

## Data Availability

The sequence data accession number for DI290 is HM016526.1 GI: 295702064). All other data generated or analyzed during this study are included in this published article and its supplementary data files and information. Any remaining information can be obtained from the corresponding author upon reasonable request.
