# Supplementary material for: Dengue virus-free defective interfering particles have potent and broad anti-dengue virus activity
Source: Commun Biol. 2021 May 11;4:557. doi: 10.1038/s42003-021-02064-7 (PMC8113447; doi:10.1038/s42003-021-02064-7)
Supplement: Supplementary file 1 — Supplementary Information [file 42003_2021_2064_MOESM1_ESM.pdf]

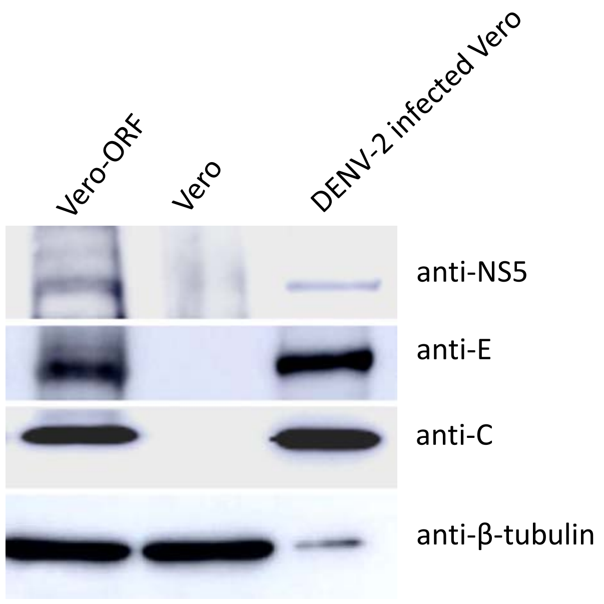

Supplementary Figure 1. Vero-ORF cells express DENV-2 proteins. Western blot analysis of cell lysates made from Vero-ORF, Vero, or DENV-2 infected Vero cells. The anti-DENV antibodies shown were used as detect DENV S and NS proteins in the cells. An anti-β-tubulin antibody was used to indicated the relative level of protein loaded into each lane.

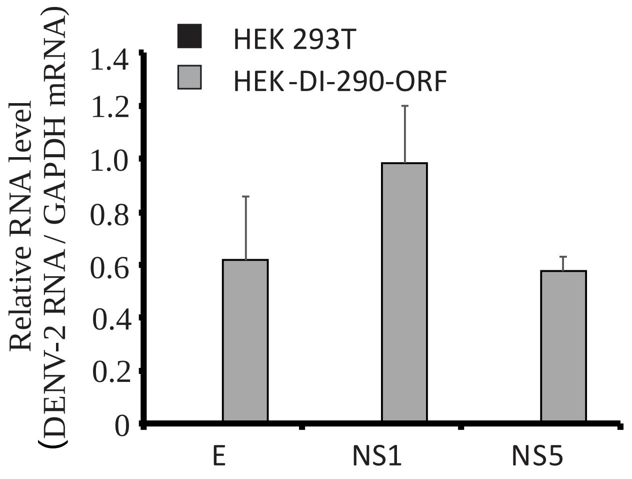

Supplementary Figure 2. Relative levels of codon optimized DENV RNAs in HEK-DI-290-ORF cells. Triplicate samples of RNA was purified from HEK 293T or HEK-DI-290-ORF cells. The RNA was analyzed by RT-qPCR using primers to detect codon optimized E, NS1 or NS5 regions of the RNAs. The levels of GAPDH were measured using specific primers and this values was used to normalize the relative level of DENV mRNA in each sample.

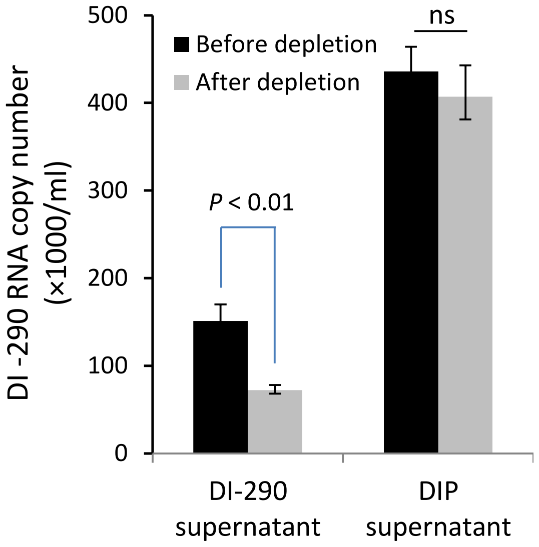

Supplementary Figure 3: Depletion of DI-290 RNA in supernatant using an anti-CD63 antibody. HEK-DI-290-ORF and HEK-DI2-90 cell culture supernatants were incubated with an anti-CD63 antibody coupled to Dynabeads to bind exosomes and RNA was extracted from depleted supernatants. Levels of DI-290 RNA detected by RT-qPCR were reduced in the HEK-DI-290 sample when exosomes were depleted, while this treatment had no effect on DI-290 RNA levels in the DIP sample.

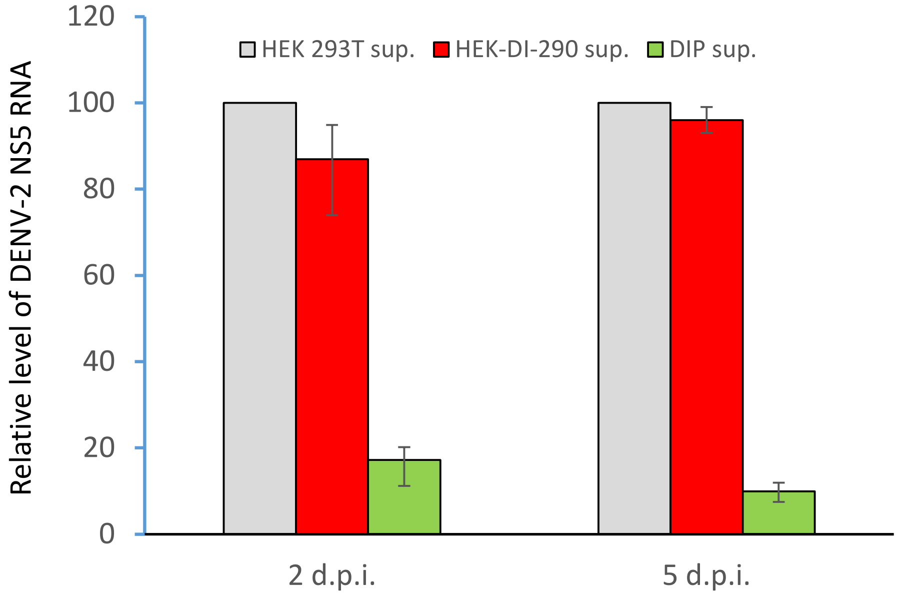

Supplementary Figure 4. Denv-2 replication in Vero cells is inhibited by DIPs. The infection of Vero cells and treatment are identical to that described for Figure 5A)

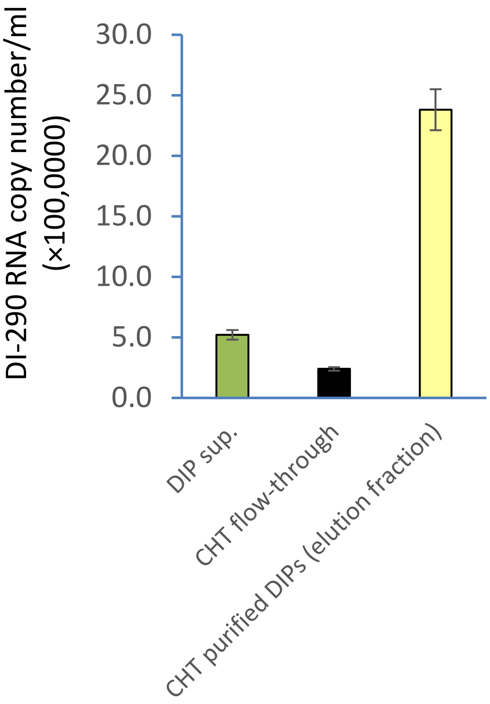

Supplementary Figure 5. DIP purification by CHT chromatography. The level of DI-290 RNA was measured by RT-qPCR in DIP supernatant, CHT flow-through and CHT purified DIPs (elution fraction). Each assay was performed in triplicate. Mean values are shown and error bars indicate the SD.
