# Supplementary material for: Dengue virus-free defective interfering particles have potent and broad anti-dengue virus activity
Source: Commun Biol. 2021 May 11;4:557. doi: 10.1038/s42003-021-02064-7 (PMC8113447; doi:10.1038/s42003-021-02064-7)
Supplement: Supplementary file 2 — Description of Additional Supplementary Files [file 42003_2021_2064_MOESM2_ESM.pdf]

Description of Additional Supplementary Files

File Name: Supplementary Data 1

Description: All source data used underlying each figure
