# Supplementary material for: Dengue virus-free defective interfering particles have potent and broad anti-dengue virus activity
Source: Commun Biol. 2021 May 11;4:557. doi: 10.1038/s42003-021-02064-7 (PMC8113447; doi:10.1038/s42003-021-02064-7)
Supplement: Supplementary file 4 — Reporting Summary [file 42003_2021_2064_MOESM4_ESM.pdf]

nature research

Corresponding author(s): David Harrich
Last updated by author(s): 18/03/2021

Reporting Summary

Nature Research wishes to improve the reproducibility of the work that we publish. This form provides structure for consistency and transparency in reporting. For further information on Nature Research policies, see our Editorial Policies and the Editorial Policy Checklist.

Statistics

For all statistical analyses, confirm that the following items are present in the figure legend, table legend, main text, or Methods section.

n/a	Confirmed	
☐	☒	The exact sample size (*n*) for each experimental group/condition, given as a discrete number and unit of measurement
☐	☒	A statement on whether measurements were taken from distinct samples or whether the same sample was measured repeatedly
☐	☒	The statistical test(s) used AND whether they are one- or two-sided<br>*Only common tests should be described solely by name; describe more complex techniques in the Methods section.*
☒	☐	A description of all covariates tested
☒	☐	A description of any assumptions or corrections, such as tests of normality and adjustment for multiple comparisons
☐	☒	A full description of the statistical parameters including central tendency (e.g. means) or other basic estimates (e.g. regression coefficient) AND variation (e.g. standard deviation) or associated estimates of uncertainty (e.g. confidence intervals)
☐	☒	For null hypothesis testing, the test statistic (e.g. *F*, *t*, *r*) with confidence intervals, effect sizes, degrees of freedom and *P* value noted<br>*Give P values as exact values whenever suitable.*
☒	☐	For Bayesian analysis, information on the choice of priors and Markov chain Monte Carlo settings
☒	☐	For hierarchical and complex designs, identification of the appropriate level for tests and full reporting of outcomes
☒	☐	Estimates of effect sizes (e.g. Cohen's *d*, Pearson's *r*), indicating how they were calculated

Our web collection on statistics for biologists contains articles on many of the points above.

Software and code

Policy information about availability of computer code

Data collection	NA
Data analysis	NA

For manuscripts utilizing custom algorithms or software that are central to the research but not yet described in published literature, software must be made available to editors and reviewers. We strongly encourage code deposition in a community repository (e.g. GitHub). See the Nature Research guidelines for submitting code & software for further information.

Data

Policy information about availability of data

All manuscripts must include a data availability statement. This statement should provide the following information, where applicable:

- Accession codes, unique identifiers, or web links for publicly available datasets
- A list of figures that have associated raw data
- A description of any restrictions on data availability

Provide your data availability statement here.

Field-specific reporting

Life sciences study design

All studies must disclose on these points even when the disclosure is negative.

Sample size	Our experiments involve 2 or 3 replicates for each of at least 3 groups, yielding a minimum of 3x data points. This achieves 80% power to for an α=0.05 test to detect a difference of y standard deviations (effect size). We expect meaningful results from our experiments to yield such large effect sizes. To further reduce our chance of making type I errors, we replicate out experiment two or three times, reducing the type I error rate to 0.0025 or 0.000125 respectively. These power calculations are quite generic and guide the design of our experiments generally.
Data exclusions	No data were excluded from the analyses.
Replication	All experiments were repeated two or three times using independent assays performed in triplicate. All attempts at replication were successful.
Randomization	NA
Blinding	Group allocation were not used in this study, so blinding was not used.

Reporting for specific materials, systems and methods

We require information from authors about some types of materials, experimental systems and methods used in many studies. Here, indicate whether each material, system or method listed is relevant to your study. If you are not sure if a list item applies to your research, read the appropriate section before selecting a response.

Materials & experimental systems

n/a	Involved in the study	
☐	☒	Antibodies
☐	☒	Eukaryotic cell lines
☒	☐	Palaeontology and archaeology
☒	☐	Animals and other organisms
☒	☐	Human research participants
☒	☐	Clinical data
☒	☐	Dual use research of concern

Methods

n/a	Involved in the study	
☒	☐	ChIP-seq
☒	☐	Flow cytometry
☒	☐	MRI-based neuroimaging

Antibodies

Antibodies used	1. Rabbit anti-DENV NS3 polyclonal antibody, Sigma Aldrich Cat. No. SAB2700181<br>2. Rabbit anti-DENV E polyclonal antibody, Gene Tex, Cat No. GTX127277<br>3. Rabbit anti-DENV C polyclonal antibody, Novusbio, Cat. No. NBP2-42822<br>4. Mouse anti-dsRNA monoclonal antibody J2, SCICONS, Cat. No. 10010200<br>5. Goat anti-Rabbit antibody, Alexa Fluor 647, Thermo Fisher Scientific, Cat. No. A27040<br>6. Goat anti-mouse antibody, Cyanine5, Thermo Fisher Sciemtific, Cat. No. A10524<br>7. Mouse anti-DENV NS5 monoclonal antibody, Clone GT361, Gene Tex, Cat. No. GTX629447<br>8. Mouse anti-beta-tubulin monoclonal antibody, clone AA2, Sigma Aldrich, Cat. No. T8328<br>9. Goat anti-rabbit IgG, horseradish peroxidase, Cell Signaling Technology, Cat. No. 7074S<br>10. Horse anti-mouse IgG, horseradish peroixidase, Cell Signaling Technology, Cat. No. 7076S
Validation	All antibodies are supplied with specification sheets from the manufacturer and validated by experiments in the report.

Eukaryotic cell lines

Policy information about cell lines

Cell line source(s)	1. HEK 293T, 293T ATCC® CRL-3216<br>2. Huh-7, JCR cell Bank, JCRB0403<br>3. Vero clone E6, ATCC C1008
Authentication	The cell lines used were not authenticated
Mycoplasma contamination	All the cell lines used tested negative for mycoplasma
Commonly misidentified lines (See ICLAC register)	NA
